# Influence of the design of modern subperiosteal multivectorial anchored Implants on success and survival in complex patient cases

**DOI:** 10.1038/s41598-025-31342-1

**Published:** 2025-12-08

**Authors:** Philipp-Cornelius Pott, Paula Schaefer-Dreyer, Michael Eisenburger, Björn Rahlf, Philippe Korn, Nils Claudius Gellrich, Meike Stiesch

**Affiliations:** 1 Department of Prosthetic Dentistry and Biomedical Materials Research, Hannover Medical School, Carl-Neuberg-Str. 1, 30625 Hannover, Germany; 2 Department of Oral and Maxillofacial Surgery, Hannover Medical School, Carl-Neuberg-Str. 1, 30625 Hannover, Germany

**Keywords:** IPS-implant, Subperiosteal implant, Success, Survival, Complications, Clinical trials, Design, synthesis and processing

## Abstract

This study investigates factors relating to the success and survival of patient-specific subperiosteally multivectorial anchored implants (IPS-Implant) with a minimum of two coupling elements (CE) to anchor the prosthetic restoration in challenging patient situations. Construction parameters of the implants were analyzed. Implantation timepoint, complications, and complication severity using USPHS criteria (A–D) were obtained from patients records. CE-location, spacing, length and transmucosal height were evaluated for their influence. The medial plaque (mPI) and the gingival (GI) indices were evaluated in order to assess inflammations around the CE. Success and survival rates were analyzed via Kaplan–Meier analysis and log-rank tests. In fifteen patients (6 men, 9 women) aged 40 to 92 in total 49 CE were examined. Kaplan–Meier analysis revealed a 2-year success rate of 91.6% and survival rate of 97.5%, while long-term analysis showed a success rate of 53.9% and survival rate of 89.4% after 8.9 years in total. Only 3 CE got lost due to fracture of the supporting bone. 46 CE are still in clinical use. After 1.8 years in the earliest, in 18 cases, signs for inflammation were detected. 10 of these inflammations required medical care. Subperiosteal implants show good short-term results but often face soft-tissue complications over time. Neighboring natural teeth and distances between coupling elements < 10 mm increased the risk for complications. Implant design, surface finish, and prosthetic protection are crucial for long-term success, requiring further research.

## Introduction

Dental implants, especially combined with backwards planning, allow successful patient treatment even in difficult situations e.g. after reconstructive surgery. Dental rehabilitation in particular with partial jaw resection often results in compromised solutions. Similar challenges also arise in patients with severe atrophy of the jaws. These cases often show a problematic soft tissue situation and large vertical distance between the occlusal plane and the implant shoulder. This results in a long lever arm and is associated with high mechanical load on bone, implant, abutment and denture. In these situations, long-term prognosis of restorations on conventional implants can be reduced. Zygoma implants can be an alternative in the upper jaw, but they are also associated with various risks of complications and the prosthetic restoration often involves compromises^1^.

**Fig. 1:**
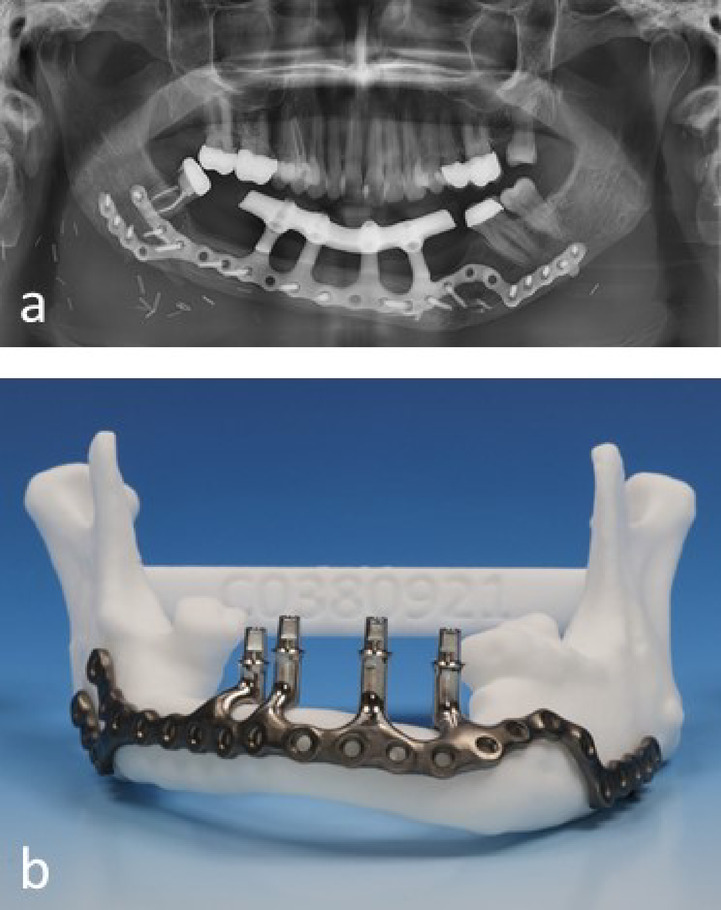
(**a**) Orthopantomograph with an IPS Implant in the patients lower jaw. An individual bar is screwed onto the coupling elements to anchor a removable denture. (**b**) IPS Implant of the patient shown in Figs. 1a and 2 on a 3D-printed model.

Interest in subperiosteally anchored implants has risen again during the recent years. Many authors have concluded that such systems offer an alternative to conventional restorations with endosseous implants in complex and difficult patient cases^2–9^. The Department of Oral and Maxillofacial Surgery at Hannover Medical School has developed a patient-specific subperiosteally multivectoral anchored implant (IPS Implant (R) Preprosthetic, KLS-Martin, Tuttlingen, Germany). This implant system combines an individual design of implant framework with standardized geometry of coupling elements (CE) to a prosthetic supra structure^10,11^. These CE can be positioned individually on the framework. Each implant has at least two CE to anchor the prosthetic restoration. The CE consist of a standardized abutment part and an individual transmucosal pillar part that connects to the implant framework. The system is anchored multivectorial in the area of stable bone structures via an individually placed framework with multiple screws. The IPS-Implant therefore allows dental rehabilitation of patients, when conventional implants are no longer an option. The main advantage is that the implant is fixed mainly in healthy residual bone located away from bony flaps and from the restoration (Fig. 1).

**Fig. 2:**
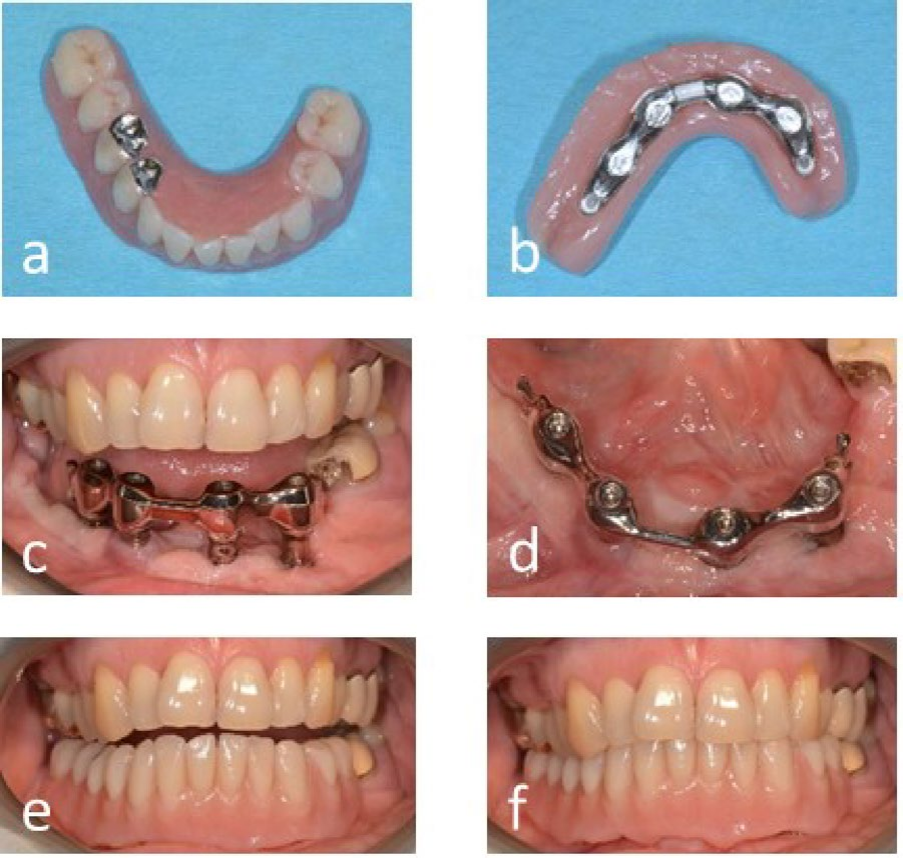
Clinical situation of the patient shown in Fig. 1a, b with and without the final denture in place. (**a**) Top view of the denture. (**b**) Bottom view of the denture with bar rider. (**c**) Intraoral situation with bar. (**d**) Top view of the bar. (**e**) Inserted denture with complete covering of the retention elements and the coupling elements. f: denture in occlusion.

FE analyses have shown that the biomechanical behavior of titanium alloys in comparison to other materials helps to reduce the load on the bone, implant screws and implant abutments in subperiosteal implants^12,13^. At Hannover Medical School, IPS-Implants usually carry a bar-supported partial or full denture. It is assumed that the saddle parts of the denture protect the coupling elements perforating the mucosa from mechanical irritations and food impaction (Fig. 2).

A follow-up study of IPS implants in the maxilla published in 2022 by Korn et al. showed that only treatable complications such as screw fractures, local inflammation and local framework exposure had occurred during the first month of clinical use. None of these complications led to implant or restoration loss^14^. In 2024, Gellrich et al. published a study with a follow-up period of up to 68 months. Similarly, no implant loss was detected and no peri-implant inflammation or screw loosening was observed^15^.

Periimplantitis is a typical complication at dental implants mostly caused by ineffective plaque removal. El-Sawy and Hegazy conclude in their review from 2024 that subperiosteal implants are a promising treatment method alternatively to conventional implants. They emphasize that the prevention of peri-implant inflammations requires special attention. In particular, they cite implant design and indication as potential risk factors^6^. Jia and Yang also address possible biological complications. They investigated biofilm development on subperiosteal implants depending on the design of a fixed denture and concluded that the basal design should be flat or oval and that the transmucosal height of the implant abutments plays a significant role in biofilm deposition^16^. Thereby, the risk of inflammation increases with higher transmucosal height. In addition to the surgical procedure, the correct indication and the choice of the implant system as well as the restoration also play important roles in the long-term success of implant-supported restorations^17^. Hygiene can be improved compared to bar-supported prosthetics on conventional implant systems by a greater distance between screw-retained anchoring elements and the mucosa. This results in a better access for oral hygiene at home, which might reduce the risk of peri-implant inflammation when the IPS implant is used.

Data on the influence of the design of subperiosteal implants on their success is still very limited. The aim of this retrospective analysis was to evaluate complications and to identify possible influencing factors on the success of IPS implants. In particular, this study evaluates differences in the design of the implant in combination with bar-retained prosthetic restorations.

## Materials and methods

The patients included were recruited during their regular follow-up appointments at the Department for Prosthetic Dentistry and Biomedical Materials Research. Hannover Medical School’s ethics committee approved the study design (Nr. 9477_B0_K_2020). All research was performed in accordance with relevant guidelines and regulations. The study protocol was in accordance with the Helsinki Declaration. Informed consent was obtained from all participants and/or their legal guardian(s). All study participants have given their written consent to participate in the study. The relevant documents are available at the clinic conducting the study.

For each patient the dates of IPS implantation and of the first complication were taken from the patient’s treatment documentation. Biological and technical complications rated by USPHS (United States Public Healthcare System) criteria in A—no treatment necessity because of complication, B—minimal complication, C—medium complication with necessity for medical care, and D—severe complication with loss of the coupling element. The influence of the localization of the coupling element, distances between neighboring coupling elements, distances between coupling element and mucosa, height of the CE pillars, the transmucosal height and the type of surrounding soft tissue were recorded.

**Fig. 3:**
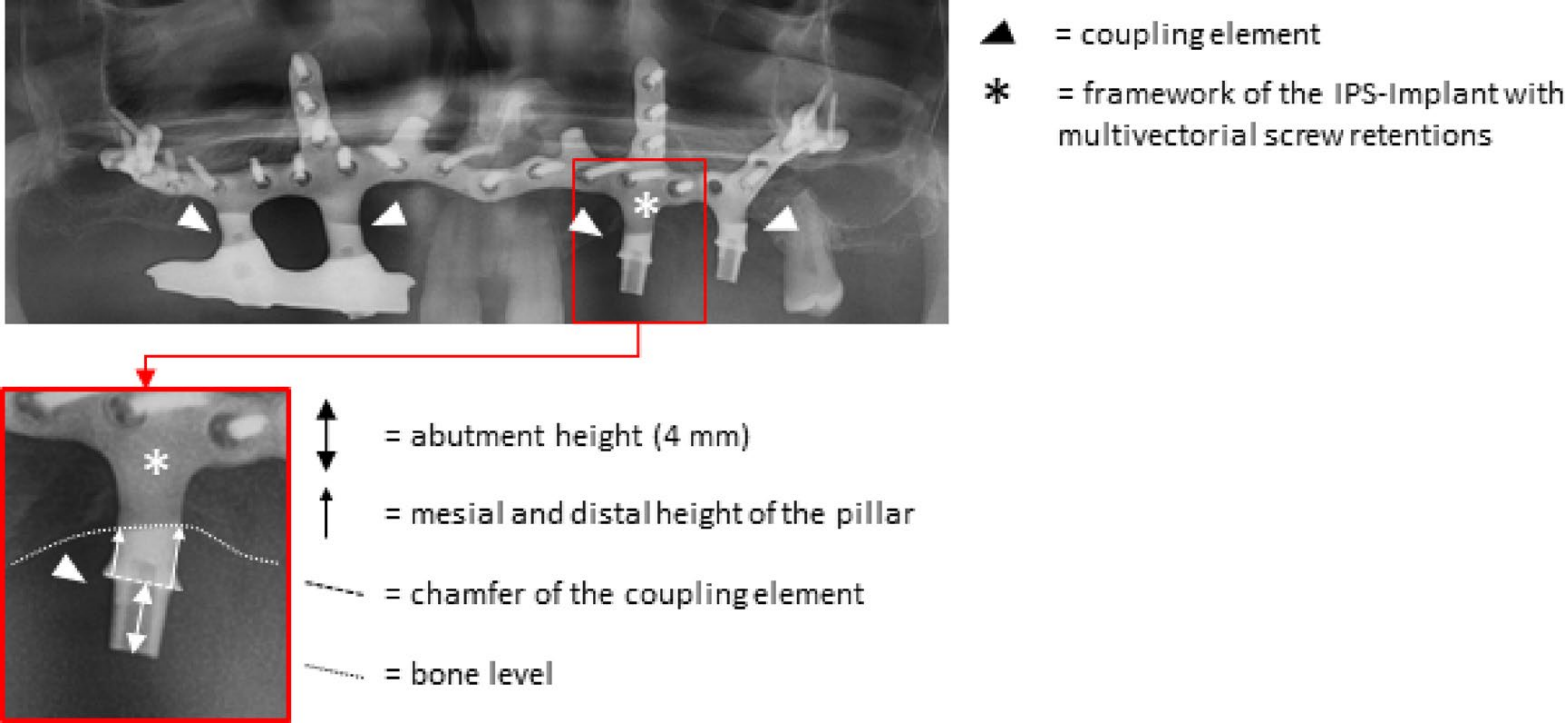
Section of a radiograph with an IPS-Implant in the upper jaw. One side shows the blank coupling elements, while a bar has already been screwed onto coupling elements on the other side. The coupling elements are marked with triangles. The respective measuring sections are also highlighted.

The length of the IPS-Implant coupling elements was measured in the postoperative orthopantomographs using a suitable software (byzz software, orangedental GmbH & Co.Kg, Bierach an der Riß, Germany). All abutments of the coupling elements had a set height of 4 mm, which was constant in all patients because of implant production. This was used as a reference value to compensate the geometrical magnification of the radiograph. The distance was measured at the mesial and distal chamfer of the CE to the bone level (Fig. 3). One experienced clinician (PSD) performed the radiographic measurements. A second experienced clinician (PCP) controlled these.

**Fig. 4:**
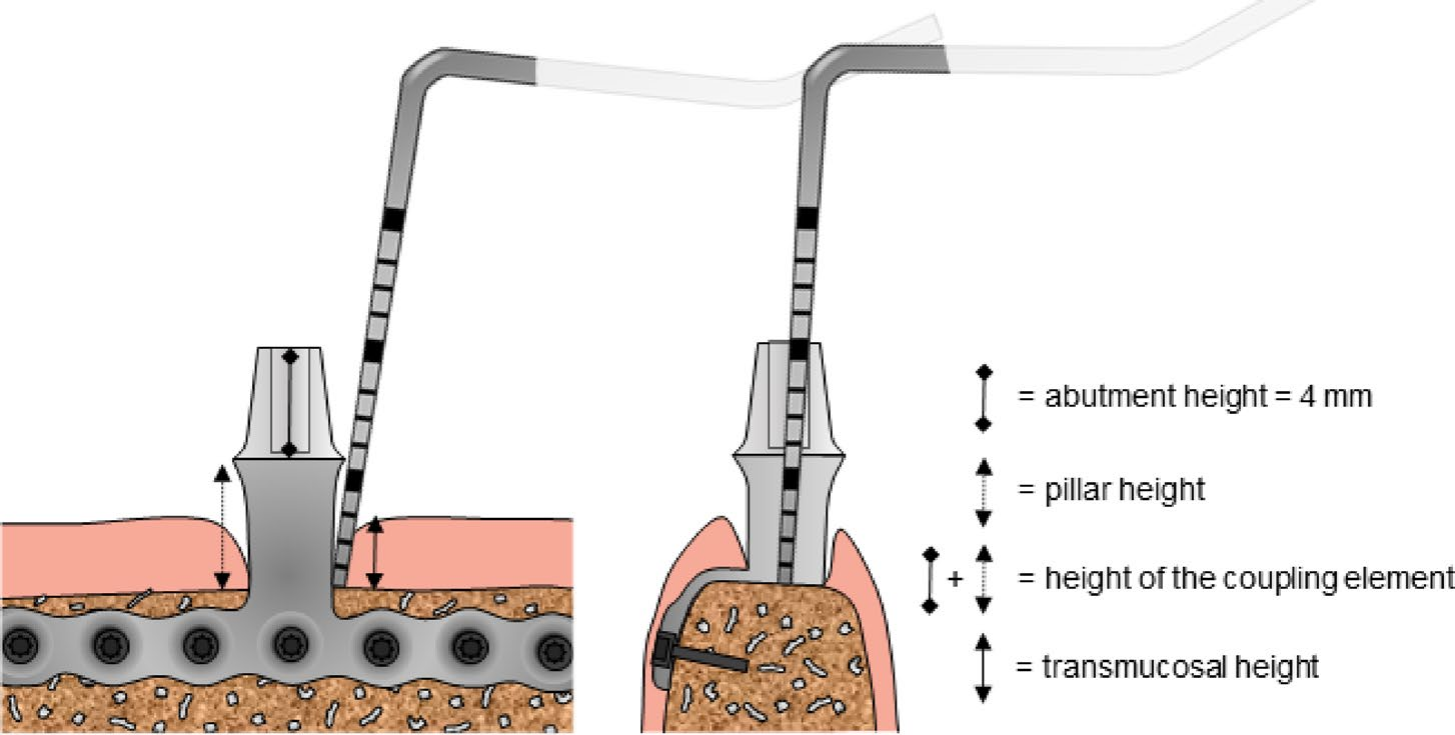
Schematic drawing of the IPS-Implant in mesio-distal and vestibulo-oral orientation. Abutment height, height of the pillar, height of the coupling element in total and transmucosal height are highlighted by markings.

A single examiner (PSD) performed all of the clinical examinations. The transmucosal height was measured clinically. It was measured at four measuring points per CE (mesio-buccal, disto-buccal, mesio-lingual/palatinal, disto-lingual/palatinal) using a standardized periodontal probe (CP15 Probe, Hu-Friedy Mfg. Co. LLC, Chicago, USA) and the deepest measurement was recorded (Fig. 4).

Medial plaque index (mPI) index was used to assess plaque accumulation^18^. This index differentiates four categories: no detection of plaque (mPI_0_), plaque is detected during probing on the smooth marginal surface of the implant (mPI_1_), plaque can be detected with the naked eye (mPI_2_) and abundant soft substance (mPI_3_). To assess marginal mucosal conditions, gingival index (GI) was used^19^. It differentiates into four categories: normal gingiva (GI_0_); mild inflammation with slight change in color, slight edema, no bleeding on probing (GI_1_); moderate inflammation with redness, edema and glazing, bleeding on probing (GI_2_); severe inflammation with marked redness and edema, ulceration, tendency to spontaneous bleeding (GI_3_). For the statistical analysis, those findings were classified as USPHS-C inflammations requiring treatment that showed pronounced signs of inflammation corresponding to GI_2_ or GI_3_. Success and survival analyses were performed with Kaplan–Meier Analysis. Groups were compared using log-rank tests. The level of significance was set at *p* = 0.05 (SPSS 29.0.1.0, IBM, Armonk, NY, USA).

## Results

In total 15 patients (6 male/9 female), aged between 40 and 92 years, agreed to participate in this study. These patients had received each one IPS-Implant with a different number of coupling elements, resulting in 49 individually analyzed measuring areas. 33 coupling elements were located in the upper jaw, 15 CE belonged to IPS-Implants in the mandible. 24 CE located in the anterior region, 25 CE were located in the posterior region. The shortest observation period was 202 days, the longest 8.9 years. Table 1 gives individual information about the patients implants.

**Table 1:** General information the reason for the implantation, previous radiotherapy and anchorage of the implant.

Patient	Jaw	Reason for IPS-implant	Radiotherapy	Anchorage of the IPS
1	Upper	Severe bone atrophy	No	Residual bone
2	Lower	Tumor	No information	Residual bone
3	Upper	Tumor	Yes	Residual bone
4	Upper	Severe bone atrophy	No	Residual bone
5	Upper	Tumor	Yes	Residual bone
6	Upper	Severe bone atrophy	No	Residual bone
7	Lower	Tumor	Yes	Residual bone, bony flap (fibula)
8	Upper	Tumor	No information	Residual bone
9	Lower	Tumor	No information	Residual bone
10	Lower	Tumor	No information	Residual bone
11	Upper	Severe bone atrophy	No	Residual bone
12	Lower	Tumor	No information	Residual bone
13	Upper	Severe bone atrophy	No	Residual bone
14	Upper	Tumor	No information	Residual bone
15	Both	Tumor	Yes	Residual bone

At 34 CE there was no necessity for any treatment (USPHS-A). Minor irritations of the marginal gingiva without inflammation (USPHS-B) occurred at 4 CE. Complications requiring medical care (USPHS-C) were observed in 8 cases, including two coupling elements with a soft tissue recession down to the implant framework and three elements with a granuloma. Fatal complications (USPHS-D) occurred in only three cases: one CE had to be removed due to a fracture of the lower jaw 52 months after implantation, but the IPS-Implant with two remaining coupling elements is still in use. After 4.5 years of clinical use, two further CE losses were documented due to partial IPS-Implant fractures also correlated to a fracture of the mandible.

**Fig. 5:**
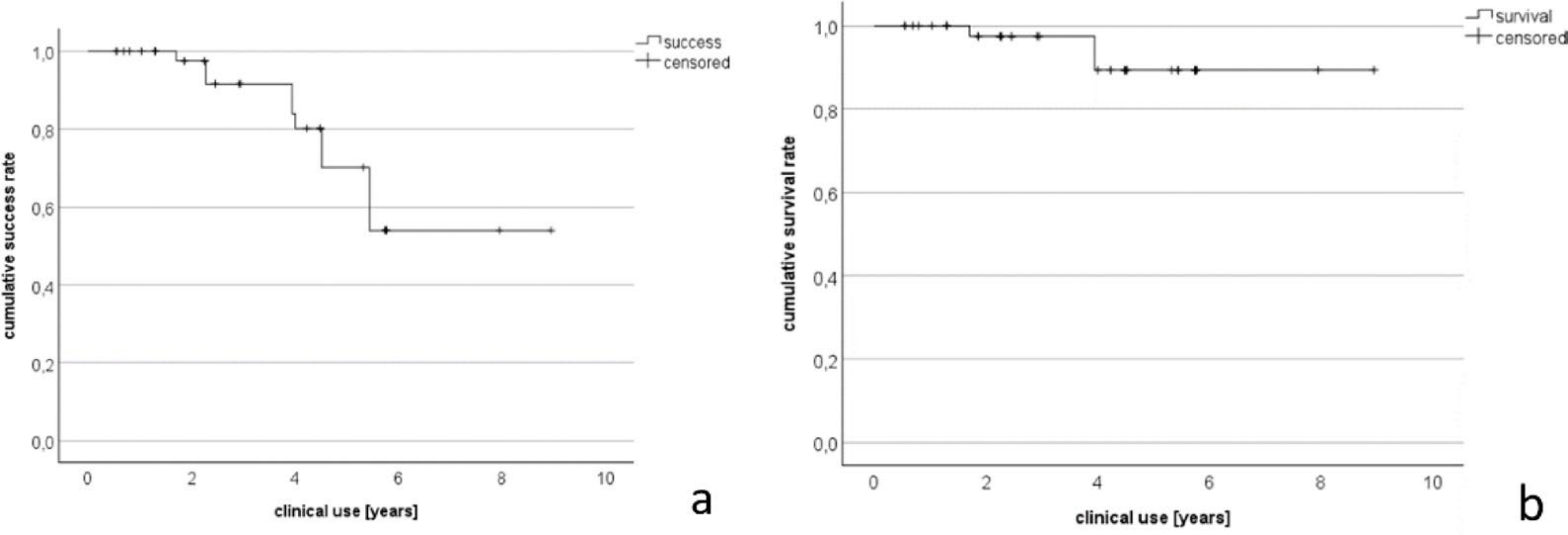
(**a**, **b**) Kaplan–Meier graphs of the success (left) and survival (right) rates over the entire observation period.

According to the Kaplan–Meier analysis, the complication analysis results in a success rate of 91.6% and a survival rate of 97.5% after the first 2 years of clinical use. Taking into account the complications with treatment necessity belonging to USPHS C and D, the success rate was 53.9% and the survival rate was 89.4% for all observed coupling elements over the entire observation period of 8.9 years (Fig. 5a, b). The Kaplan–Meier estimator for complication-free time was about 6.8 years (SD 0.5 years, CI 5.8–7.7 years). With regard to survival time, the result was 8.3 years (SD 0.3 years, CI 7.7–9.0 years).

**Fig. 6:**
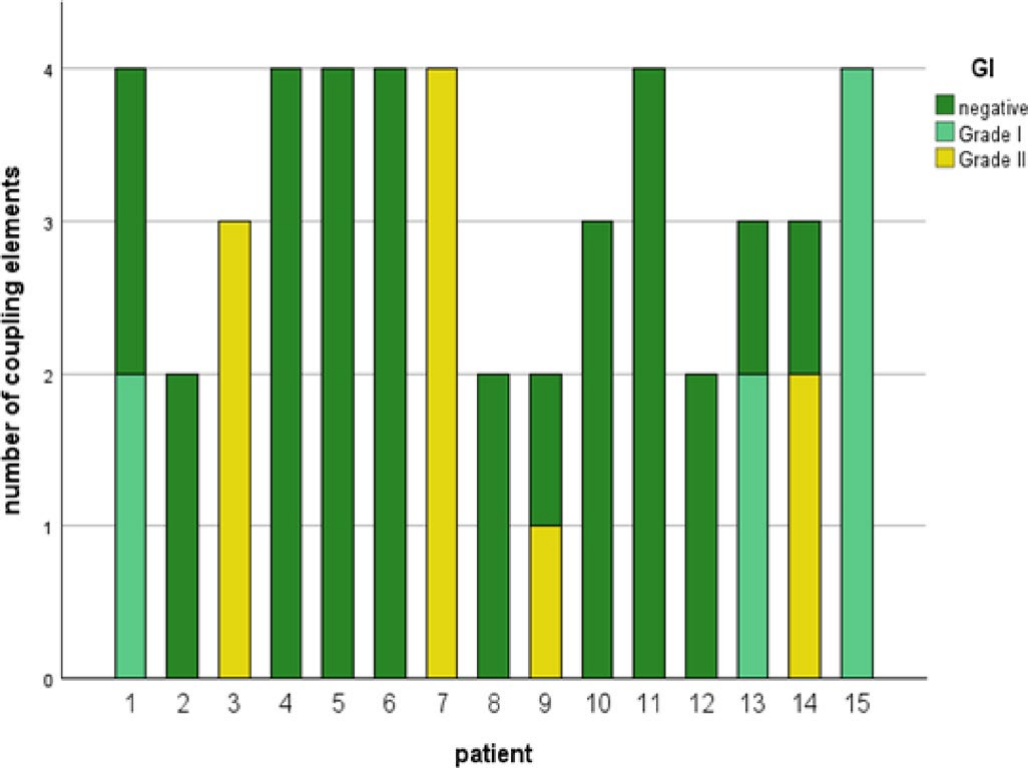
GI distribution in each patient. dark green = negative; light green = grade I, yellow = grade II.

**Fig. 7:**
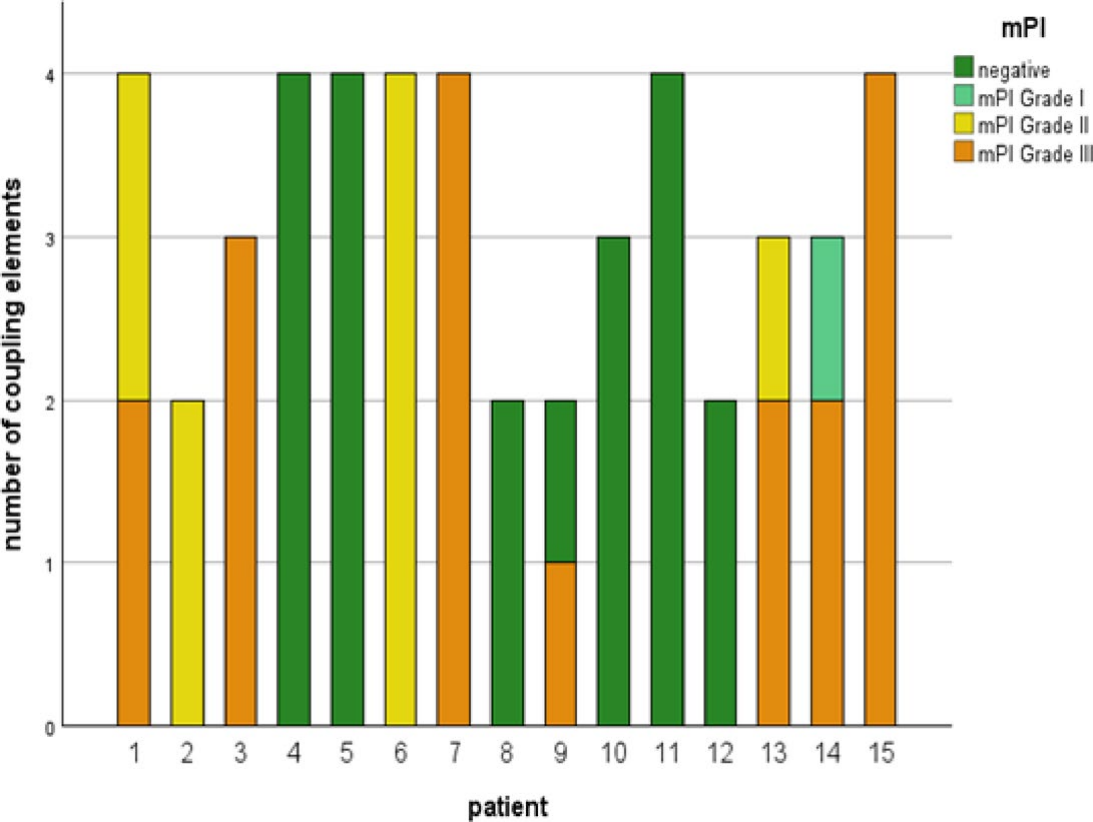
mPI distribution in each patient, dark green = negative; light green = grade I, yellow = grade II, orange = grade III.

Due to the highly vulnerable patients with a generally increased risk of inflammation, in this study, the inflammations were considered separately. The first inflammations occurred after at least 2 years of wear. In total 18 CE showed signs of inflammation. Initial signs of inflammation without treatment necessity (GI_1_) occurred in the surrounding of 8 coupling elements. Inflammation requiring medical care (GI_2_) occurred at 10 CE in four patients (Fig. 6). At all of these 18 coupling elements, biofilm formation was detected in accordance with mPI_3_ (Fig. 7).

The success rates referring to the influence factors and the corresponding *p*-values are given in Table 2.

**Table 2:** Overview about the influencing factors with their individual categories, number of coupling elements belonging to the categories in total, success-rates in percent, and *p*-values are given.

Factor		Total	Success (%)	*p*-value
Jaw	Upper	33	69.1	0.834
	Lower	16	59.1	
Localization	Anterior	24	64.5	0.733
	Posterior	25	63.9	
IPS in combination with	Dental implants^a^	15	93.3	**0.005**^**a**^ ≥ 0.155
	Natural teeth^a^	23	40.0	
	Dental implants and natural teeth	11	90.9	
Surrounding tissue	Oral mucosa	27	76.5	0.172
	Transplant	22	52.5	
Distance between coupling elements	< 10 mm^bc^	4	0.0	**0.018**^**b**^ **0.025**^**c**^
	10–14 mm	23	62.8	
	15–19 mm^c^	18	72.7	
	≥ 20 mm^b^	4	100	
Pillar heigth in total	< 2.5	15	77.0	≥ 0.101
	2.5–5.0	18	46.7	
	5.0–7.5	7	80.0	
	> 7.5	9	57.1	
Distance IPS abutment to marginal gingiva	0	21	57.9	≥ 0.343
	< 0.5	16	73.1	
	< 1.0	8	75.0	
	> 1.0	4	100	
Transmucosal height	< 1	6	83.3	≥ 0.082
	< 3	17	79.1	
	< 6	13	42.0	
	> 6	13	72.7	

Significant pairings are marked with superscript letters.

Significant values are in [bold].

## Discussion

Patient-specific subperiosteal implants can be used for implant-supported prosthetics if conventional implants are not suitable any longer. This can be seen particulary in patients where treatment with conventional implants in combination with bone augmentation has already failed. Another indication for IPS-Implants is severe loss of bone as it can be found in patients who have undergone tumor surgery. In these situations, IPS-Implants can offer the last attempt for implantation and prosthetic reconstruction. These patients often show a severely reduced wound healing capacity and a high risk for peri-implant complications, especially for soft tissue inflammations. The customized 3D design of the IPS implants and their production using computer-aided additive manufacturing processes can improve the compromise situation described above in complex patient cases to the effect that the mechanical load on the prosthetic anchorage components shifts closer to the masticatory plane. This leads to an idealization of the lever ratios, reduces load on anchorage elements and thus improves the long-term prognosis of the restoration compared to situations with conventional implants. In this study, only three fractures of IPS implants occurred. These were related to traumatic mandibular fractures and not to mechanical overloading of the restoration or implant. Other typical loading-associated complications, such as screw loosening, did not occur.

In particular, the high risk of peri-implant inflammation in this highly vulnerable patient group must be given special consideration when interpreting the data of the presented study. For this reason, a comparison with studies on the success and survival of conventional implants is only possible to a very limited extent. Unfortunately, the international data on the success of modern subperiosteal dental implants is still extremely limited. In 1993, Stvrtecky and colleagues retrospectively analyzed the success of subperiosteal implants over a period of 15 years. They found a survival rate of 58.3% after 10 years. They conclude that the success rates in the mandible were higher than in the maxilla^20^. In the present study, the survival rate of the IPS-implants after 8.9 years of clinical use was 89.4%. In particular, success and survival rates after 2 years of 91.6% and 97.5% demonstrate the potential of the modern IPS-Implants examined in this study. When comparing the data to the literature, it must be taken into account that the oldest implants examined by Stvrtecky’s group had been inserted as early as 1978. Since then, design and manufacturing techniques in particular have developed to such an extent that modern implant systems are superior to these old systems in many ways. Modern CAD software enables patient-specific planning of complex three-dimensional structures based on 3D radiographs, which can be supplemented by scan data from models or direct intraoral scans using best-fit matching. Computer-aided manufacturing (CAM) of high-performance alloys such as Ti6Al4V can now also be carried out using selective laser melting (SLM). This CAD/CAM processes enables to produce the implants even in a complex shape^6,11^. The patient-specific design of the IPS-Implants allows for remote and multivectorial anchoring with mini-screws, which ensures a stable load-bearing situation immediately after implantation. This is a huge difference to old subperiosteal implant systems, which had to be bent onto the local bone in a saddle shape and were only held in position by the adapted periosteum with the overlying mucosa. Comparable to the presented data, Strappa et al. found no complications up to a 2 year follow up in their study about additively manufactured subperiostal implants from 2022^21^. In their 2024 review, Anitua et al. summarized 13 articles including 227 implants and concluded that modern subperiostal implants showed few complications during the first few years. However, they emphasized that in some cases, complications associated with soft tissue, especially inflammation, may occur over time^22^. These results are also consistent with the data presented in this study. After the first 2 years complications mostly associated with the soft tissue. A possible reason for the occurrence of complications could be the design of the implant itself and the design of the restorations. According to Aniuta et al., it seems not to be important whether a fixed denture was screwed or cemented onto the implant^22^. However, according to the author’s knowledge, the influence of removable dentures on possible complications has not yet been addressed in previous publications.

In general, the design of the dental prosthesis itself plays an important role in the occurrence of complications with implants. In particular, a design that allows good oral hygiene helps to minimize bacterial biofilm accumulation reduces the risk of peri-implant inflammation^23^. In accordance with the treatment concept for IPS-implants of Hannover Medical School, patients included in this study were treated with bar-supported removable dentures. The bars allowed an easy access to the IPS CE for daily oral hygiene. Nevertheless, this study also found soft-tissue-associated complications around 18 of 49 CE. All CE with signs of inflammation (GI_1_ and GI_2_) also showed pronounced biofilm attachment (mPI_3_). These indicate ineffective oral hygiene at the individual patient level, which in this case was not due to the IPS implant or the design of the restorations.

The investigation of various influencing factors showed a significant influence of the distance between the CE on the development of complications. It was shown that the greater the distance between the CE, the less complications occurred. The distance should not decrease below 10 mm (Table 1). This aspect has not yet been investigated. Therefore, no comparative literature could be identified.

Furthermore, data showed that the success rate was significantly lower when natural teeth were located in the direct neighborhood of the coupling elements. A possible reason for a bacterial colonization at the CE could be the comparable colonization of teeth and implants with biofilm deriving from periodontal pockets^24^. Particularly in patients who have an increased risk of periodontal inflammation, the risk of peri-implant inflammation is also increased. This is particularly important for the highly vulnerable patient groups cared for here. Interestingly, the combination of subperiosteal implants with conventional implants showed no influence on the occurrence of complications (Table 1).

Furthermore, the transmucosal height trends to have an influence on the occurrence of complications. In the patient group included in the presented study, more complications occurred at a transmucosal height between 3 and 6 mm than at higher or lower values. However, this difference was just not statistically significant (Table 1). This tendency is supported by the data of Jia and Yang, who found a significant influence of transmucosal height on the attachment of biofilm to subperiosteal implants restored with bridges^16^. In the presented study, it remains unclear why there were more complications at transmucosal heights between 3 and 6 mm. It would be more reasonable to assume that the number of complications observed increases with increasing probing depth and thus poorer oral hygiene at home. There may be a connection to the design of the denture, which in the present study was basically a bar-supported removable prosthesis. The coupling elements were covered up to the mucosa by the denture base. This reduces the possibility of food impaction, which helps to prevent biofilm.

The implants examined in this study were all anchored multivectorially to healthy bone using osteosynthesis screws. One implant was additionally attached to a fibula graft after partial resection of the lower jaw. Accordingly, this study cannot clarify the influence of the bone bed on the success rate of subperiosteal implants. The data collected here is also insufficient to determine the influence of radiation on the occurrence of complications. Accordingly, further studies are necessary to clarify these issues.

To evaluate the influence of the biofilm on the inflammation processes, in this study the GI and mPI values were considered together for each patient. All CE showing GI_2_ also showed mPI_3_. There were also patients in whom biofilm was detected on CE but no pronounced inflammation was diagnosed. The results of the presented study underline the importance of the surface quality of the transmucosal parts of the CE. Based on the results, it must be concluded that a high-gloss surface is highly important for these parts to reduce the biofilm adhesion. Since the data presented here do not come from a prospective follow-up study, it cannot be ruled out that inflammations of the mucosa may develop over time. In this context, further data are needed which will be collected in the future.

## Conclusion

Taking into account the limitations of this study, the following conclusions can be drawn: The subperiosteal IPS-Implants examined here show very good results in the first years. However, during a longer period primarily soft-tissue-associated complications occurred. Fatal complications are rather rare. The design of the IPS-Implant structure itself plays a role in long-term success. The distance between coupling elements should not be less than 10 mm and immediate proximity to natural teeth should be avoided. A high-gloss surface finish is important for the transmucosal parts of the IPS-Implants. It is assumed that a removable dental prosthesis protects the areas where the IPS-Implant CE pass through the mucosa and might help to prevent biofilm on the subperiosteal implant framework. Nevertheless, patients with subperiosteal implants should have professional tooth and implant cleanings performed 3–4 times a year in addition to regular oral hygiene at home to minimize the risk of peri-implant inflammation. Further research is needed to increase the data of long term success and to identify further factors, which might influence the success and survival of these IPS-Implants.

## Data Availability

The datasets used and/or analysed during the current study are available from the corresponding author on reasonable request.

## Abbreviations

CAD/CAM: Computer aided design/Computer aided manufacturing
CE: Coupling element
IPS-Implant: Patient specific implant
GI: Gingiva index
mPI: Medial plaque index
USPHS: United States Public Healthcare System
